# On the Way

**DOI:** 10.1007/s00062-020-00977-0

**Published:** 2020-12-10

**Authors:** Horst Urbach

**Affiliations:** Dept. of Neuroradiology, University Medical Center Freiburg, Breisacher Straße 64, 79106 Freiburg, Germany

The 55th annual congress of the German Society of Neuroradiology was the first (and very likely not the last) congress held online. We appreciated 570 colleagues joining a 2.5-day program presented in 1 or 2 parallel streams. In order to at least partly compensate for the lack of social contacts we tried to be very interactive offering chat communications, institutional tickets, the game form Quiniela, and goodies, such as Yoga, resilience and a movie podcast.

Our industrial partners supported the meeting with symposia and presented their portfolios on a virtual marketplace. Many scientific presentations are available on demand up to 1 year after the congress. While the congress was a first amazing event, we have to develop it further keeping in mind that the success of our specialty neuroradiology has always been driven by digitalization. What began with Hounsfield’s CT in the 1970s and MRI and DSA in the 1980s, has become a new spin in recent years considering artificial intelligence (AI) applications, teleproctoring for interventions, augmented reality for imaging-assisted surgery, and others. To integrate these “digital” developments in an appropriate manner in order to teach ourselves on future congresses is a need; however, the flexibility to organize inspiring congresses in different formats in a pandemic period is the real challenge.

Prof. Dr. Horst Urbach

Klinik für Neuroradiologie

Universitätsklinikum Freiburg

Neurozentrum

